# Reconsidering the ‘meritocratic power of a college degree’

**DOI:** 10.1016/j.rssm.2020.100479

**Published:** 2020-04

**Authors:** Dirk Witteveen, Paul Attewell

**Affiliations:** a Nuffield College, University of Oxford, United Kingdom; b The Graduate Center, City University of New York, United States

**Keywords:** Parental education, Intergenerational mobility, Equalization thesis, College graduates, College selectivity, Meritocracy

## Abstract

Previous research has shown that the intergenerational transmission of advantage disappears once individuals obtain a bachelor’s degree. This is known as the equalization thesis: the ‘meritocratic power’ of a college degree. This paper revisits the question of origin-destination association among college graduates. We improve on earlier studies by using three large sample (40,000+) of the National Survey of College Graduates, consisting of birth cohorts between 1938 and 1985. Contrary to the equalization thesis, we find that parental education and parental income are associated with substantially higher post-college incomes. An individual’s own attainment only partially mediates the association through the type of college attended, but not through attaining an advanced degree. The consistency of the origin-destination estimates across three decades supports a reproduction thesis of mobility.

## 1 Introduction

Researchers contend that education plays a dual role in social stratification and mobility. On the one hand, education provides a route for the intergenerational transmission of status, because advantaged families facilitate a lengthier or superior education for their offspring that leads their descendants towards better jobs and higher incomes. “On the other hand,” Featherman and Hauser (1978, p.255) explain, “… education played a direct role … that countered the stratifying effect of the former role. Thus, the occupational handicap of birth into a low social stratum might be overcome by education…”

The relative importance of these two facets of education – the stratifying versus the mobility role – has remained of central interest for generations of researchers, in part because scholars differ concerning the balance between these two forces. Blau and Duncan’s (1967) study, replicated by Featherman and Hauser (1978), observed significant paths from father’s education and occupation to son’s occupational attainment via son’s education – the stratifying aspect.^1^ They also noted, however, that the major part of the relationship between son’s education and son’s occupation was independent of social origins or parental background, interpreting this as evidence for a trend in the United States of “declining status ascription and increasingly universalistic status allocation” (Featherman and Hauser 1978, p.481).

The belief that when Americans from lower-class origins beat the odds and complete a higher educational credential they escape their disadvantaged family origins and enjoy the same fruits from educational achievement as more advantaged collegians has further developed in subsequent decades. The general argument of this so-called *equalization thesis* is that occupational positions have become more dependent on rational selection through the educational system in highly industrialized countries, erasing the influence of family background in high-skill job matching.

Hout (1984) analyzed mobility tables from two Occupational Change in a Generation studies and observed that the association between father’s occupational status and son’s occupational status declined markedly between the 1962 and 1973 tables. Intergenerational transmission weakened yet further in General Social Survey data spanning 1972–1985 (Hout, 1988). These findings led Hout (1984, p.1404) to conclude: “origin status does not affect destination status among college graduates,” adding “This finding provides a new answer to the old question about overcoming disadvantaged origins: A college degree can do it.” Using more General Social Survey data, Hout (2018) recently confirmed that achieving a BA or higher degree erases the influence of family background – measured by parent’s occupational attainment – on offspring’s occupational attainment.

Extending beyond measures of occupational attainment, Torche (2011, 2018) tested what she termed the “meritocratic power of a college degree” using a wide variety of measures for both origin and destination (social class, socioeconomic index, occupational prestige, earnings, and family income). Despite examining a range of different measures, she concluded “… the chances of achieving economic success are independent of social background among those who attain a BA. The finding is largely consistent across all indicators of socioeconomic standing…” (Torche, 2011, p.798).^2^ Diverging somewhat from Hout’s original thesis, Torche separated individuals who went no further than the bachelor’s degree from those with advanced degrees (master’s, PhDs, and advanced professional degrees). For those with advanced degrees, she found that there was a significant relationship between social background and offspring’s outcomes, but she confirmed Hout’s “meritocratic” pattern of no relationship between parental background and offspring’s outcomes for those graduates who only obtained a bachelor’s degree. Torche (2011)) highlighted a “U-shaped” relationship between origin and offspring, as mediated by educational attainment; one that reappears in strength for advanced degree holders.

However, the equalization thesis regarding the meritocratic power of a college degree has been questioned by several recent studies (discussed below) which document significant earnings disparities for collegians related to their family background and contradict the equalization thesis. The contribution of the present paper is to add new analyses from three large nationally representative surveys of college graduates in the US that estimate intergenerational effects for cohorts born between 1938 and 1985. All three surveys provide evidence of a substantial and persistent intergenerational relationship between parents’ education and offspring’s earnings. This is evident for men and for women considered separately, and for individuals who earn no more than a bachelor’s degree as well as for those attaining higher degrees. Importantly, the intergenerational coefficients seem consistent over time and are not attributable to age or career stage.

In sum, we document longstanding and substantial intergenerational transmission of advantage among the U.S. college-educated population. We concentrate on two specific indicators of socioeconomic standing between generations: parental education and offspring’s earnings. These measures have been used in prior studies on origin-destination associations, including specific tests of the equalization thesis in the U.S. context. The review following addresses the broad hypothesis of equalization, as well as recent specific applications to U.S. data. We conclude by discussing why our results, along with those of several other recent studies, differ from earlier research in the status attainment tradition and consider what this implies for the sociological understandings of achievement, ascription, and social mobility.

## 2 Literature

### 2.1 Long-term perspectives on social mobility and higher education

Mobility research has traditionally focused on the association between parental class position and offspring’s class position. This approach assumes that a small or non-significant association between origin and destination would indicate a high level of social *fluidity* – a society in which inequality of opportunity is low – and vice versa. Measuring hierarchical positions has typically been based on either class schemas or occupational prestige scales (Breen & Jonsson, 2005), such as the Eriksson-Goldthorpe-Portocarero (EGP) schema or the Duncan Socioeconomic-Index (SEI). Furthermore, as a result of data availability of parents’ positions and the dominance of men in the workforce, origin and destination estimates have primarily relied on the father-son associations.

An important stream of social mobility research addresses the question whether intergenerational mobility estimates change *over time* – between cohorts. According to one theoretical perspective (Blau & Duncan, 1967; Treiman, 1970), the origin-destination association should steadily decrease among younger generations as a result of structural and processual changes. Highly industrialized countries should require increasingly more differentiated labor markets, creating mass-middle class societies with more space for initial upward mobility and long-term equality of opportunity. More importantly, such a continuous differentiation of the division of labor in advanced economies would also require a more rational procedure of social selection (i.e. meritocracy). As societies become more ‘open’ – less dependent on ascription and more on achievement in education – the origin-destination association would sharply decline. This perspective is called the “liberal hypothesis.”

Higher education indeed expanded in the United States and in many other advanced economies throughout the second half of the twentieth century, providing significant chances for upward mobility and social fluidity. However, contrary to the liberal hypothesis, Erikson and Goldthorpe’s cross-national study *The Constant Flux* (1992) found very little change in social class fluidity across birth cohorts in 15 countries between the 1960s and 1970s. Using US data only, Pfeffer and Hertel (2015) found only modest consecutive increases of mobility between birth cohorts of 1922 thru 1982 on this class dimension. Building on this finding, Bloome, Dyer, and Zhou (2018) showed that one important determinant of this persistence is the suppressing effect of educational expansion on the parent-offspring association. Bukodi and Goldthorpe (2019) recently returned to this question reporting that inequalities in mobility chances by social origins have not declined between the 1970s and the 2000s. Importantly, they argue that modern educational systems – including higher education expansion – have not led to the predicted increased fluidity between origin and destination. This argues for a “reproduction hypothesis” of intergenerational mobility. Erikson and Goldthorpe (1992) acknowledge Hout’s (1984, 1988) studies of the equalization hypothesis and considered the possibility that the United States might be in the process of becoming a more open society. However, they did *not* find convincing evidence for an American exception to the ‘constant flux’ based on population-wide estimates from their own class schema.

### 2.2 Recent studies on origin-education-destination

Independent of society-wide mobility, the potential role of the educational system in facilitating equalization has been addressed by numerous sociological studies concerned with educational attainment. However, a large body of research shows that academic success remains strongly associated with family background. In the United States, kindergarteners begin their schooling with substantial socio-economic status (SES)–related differences in vocabulary, numeracy, reading readiness, and general knowledge (Duncan & Murnane, 2011; Hart & Risley, 1995; von Hippel, Workman, & Downey, 2018). Initial skill disparities tend to widen as children progress from kindergarten through twelfth grade, in part due to SES-related fallbacks in skills during summer breaks (Cunha & Heckman, 2007; DiPrete & Eirich, 2006; Downey, Von Hippel, & Broh, 2004; Heyns, 1978; von Hippel et al., 2018). During middle and high school, lower income students are less likely to enroll in advanced courses, and more of them drop out of school (Kaushal, Magnuson, & Waldfogel, 2011; Lucas, 1999; Rumberger, 2004). This pattern of cumulative disadvantage extends into higher education where family SES is associated with whether a student continues on to college, the kind of institution attended (community college versus four-year, selective versus unselective college), and the likelihood of graduation with a baccalaureate degree (Bailey & Dynarski, 2011).

Two recent books on the origin-destination relationship in the United Kingdom discuss class inequality among higher-educated groups and question the meritocratic power of a college degree. First, Bukodi and Goldthorpe (2019) found a strong direct effect of social origin (parental status and education) on offspring’s class destinations that is also stable over time – between successive cohorts. The authors describe the remaining class background disadvantage among college-educated individuals in terms of a glass (or class) ceiling mechanism: a limitation of “upward mobility of children from less advantaged origins who have performed well educationally.” (p.152). Second, using similar data and analyses, yet emphasizing the post-college pathway, Friedman and Laurison (2019) documented that even when British working-class individuals attend top universities, they are still less likely to get access to top jobs, compared to middle- or upper-class graduates.

While these findings reaffirm earlier work on British and European persistence of a class gradient among college graduates, the prevailing thesis in the United States argues for a complete equalization of backgrounds among US bachelor’s degree holders – i.e. null effects of origin on destination (1988, Hout, 1984; Torche, 2011) – and holds pride of place in social science textbooks on social stratification (Grusky, 2014). In addition to social class or a socioeconomic index, researchers have examined several different measures of social origin, such as parental SES, parental income, and parental education. Despite the fact that comprehensive premise of meritocratic thesis regarding the origin and destination definitions, and the multitude of measures used by researches to test it, several recent US studies now show a substantial influence of social origin on college graduates’ labor market position.

#### 2.2.1 SES

Socioeconomic status is measured as a combination of education, income, and occupation. It therefore provides a robust summary indicator of origin socioeconomic standing. Giani (2016) examined parental SES influences on offspring’s annual earnings and wages for a sample of 2430 graduates from the national Educational Longitudinal Study, which followed a cohort of students who were high school sophomores in 2002 until 2012. Among BA recipients, he reported a difference of $5200 in annual earnings when comparing college graduates from high- and low-SES parental backgrounds. He next examined outcomes separately for colleges of different selectivity, as a mediating variable. For the most selective colleges, parental background ceased to be significant, but for moderate and non-selective colleges, significant family SES-related earnings gaps remained.

#### 2.2.2 Income

Recent social surveys have increasingly collected data on income, including that of the parental home. This measure emphasizes the potential role of family’s economic capital in explaining post-college stratification mechanisms. Gregg, Jonsson, Macmillan, and Mood (2017) analyzed data from the United States, Britain and Sweden to examine national differences in the relationship between fathers’ and sons’ incomes. For the United States, they used the National Longitudinal Survey of Youth (NLSY) that provides intergenerational data for 958 sons. They found less mobility (a stronger relationship between father’s and son’s incomes) in the United States than in the United Kingdom, and most mobility in Sweden. They also estimated the proportion of intergenerational transmission that operates through offspring’s education and found that, in the United States, most (57 percent) of transmission is not via education, in contrast to the longstanding view that posits a shift from ascription to educational achievement.

Focusing specifically on the U.S., a few studies have examined the relationship between parental income and individual earnings for college graduates. First, Witteveen and Attewell (2017b) analyzed data from two waves of the Baccalaureate and Beyond longitudinal study, one of which followed graduates for four years and the other for ten years beyond the baccalaureate. They found substantial earnings disparities associated with family background, which persisted after controlling for college selectivity, major, and academic performance. Second, Bartik and Hershbein (2018) analyzed Panel Study of Income Dynamics (PSID) data on the earnings of college graduates from lower and higher income families. They “find that career earnings of bachelor’s graduates who grew up in low-income households are substantially lower than similarly educated individuals who grew up in higher-income households” and that “the career *percentage* earnings premium from earning a bachelor’s degree, relative to only a high school diploma, is much lower for individuals who grew up in low-income families” (Bartik and Hershbein, 2018: 4). Those patterns applied to whites and to men, but not to women or African-Americans.

Also concentrating on the earnings differences of higher-educated Americans, Chetty, Friedman, Saez, Turner, and Yagan (2017) examined the role of colleges in facilitating upward intergenerational mobility by linking lists of undergraduates reported by colleges to the federal government to Internal Revenue Service (IRS) databases that relate parental to offspring earnings. They found a high correlation between parental income and offspring’s income among collegians (i.e. higher education *attendees)*. This transmission reflects a two-part process: students from higher income families are far more likely to attend a highly selective college, and students who attend those colleges earn considerably more post-college than students who attend less selective colleges. For example, a child from a top-1 % family is 77 times more likely to attend an Ivy Plus college than a child from a bottom income quintile family. Conditional on attending a particular level of college, however, the relationship between parental income and offspring’s income is only one-quarter as large as the overall intergenerational effect. Thus, parental background operates mainly via the child’s access to a particular level of college.

#### 2.2.3 Education

Finally, parental education is also frequently used as a measure of origin – as a proxy of parental resource *capacity* rather a direct measurement of such capitals. Manzoni and Streib (2018) analyzed Baccalaureate and Beyond data, focusing on the contrast between first generation collegians (whose parents did not attain a college degree) and continuing generation students (whose parents had a BA or higher degree). They reported significant post-college wage gaps between first generation and continuing graduates of 11 percent for men and 9 percent for women. They also documented substantial sorting of first-generation students into different levels of college selectivity, such that generational earnings differences among students who attend colleges of the same level of selectivity are much smaller than among the sample as a whole.

Emmons, Kent, and Ricketts (2018) utilized the Survey of Consumer Finances for 2016, finding that a white household headed by a baccalaureate whose own parents earned a college degree earned $156,756 compared to $114,225 for a similarly educated person whose parents lacked a BA. They conclude: “College clearly is important, but contrary to conventional wisdom, your own college education does not completely level the playing field. … In this comparison, inherited demographics – including the college education of the parents’ generation – outweighed the benefits of obtaining a college education” (Emmons, Kent, and Ricketts, 2018 p.13).

Ford (2018) analyzed data collected as part of the multinational OECD Program for the International Assessment of Adult Competencies (PIAAC). She compared monthly earnings of college graduates aged 25–54 whose parents had not completed a bachelor’s degree or higher (‘first generation’) with those whose parents had completed a baccalaureate. For this sample of 919 individuals, she found that first generation graduates earned about $600 less per month than those whose parents were college graduates. However, this difference ceased to be statistically significant after controlling for level of degree attained plus numeracy and other skill variables. She concluded that the effect of parental education on earnings operated primarily through differences in the level of degrees and skills that the offspring attained.

However, other scholarly work that explicitly test the meritocratic thesis in the US context have instead confirmed its equalization mechanism. These studies have primarily concentrated on socioeconomic status as their origin measure. Using the NLSY’79, Karlson (2019) uses an inverse-probability weighting approach for selection into two educational groups: college degree holders and advanced degree holders. Focused on the intergenerational SES association, the study found non-significant estimates in the models for both groups. These null findings are in support of the meritocratic thesis among *all* college educated groups (yet disputing Torche’s U-shaped relationship with regard to master’s degrees or higher).

Thompson (2019) also used the NLSY’79 to examine the SES association and the income association between parents and their children and confirmed the equalization thesis in many respects. For occupational status, any bachelor’s degree erases the impact of parental status on that of offspring (i.e. consistent null findings of social origin). However, for income attainment, null findings consistently appeared for the group of ‘less-selective bachelor’s degree’ holders, but not at the top (selective) or the bottom (nonselective), leading the author to conclude that mobility primarily operates in the middle of college selectivity spectrum.

To summarize: several studies using different datasets have found that the earnings of college graduates after they leave college differ according to various measures of parental background, and thereby cast doubt on the ‘college as equalizer’ or meritocratic thesis. Yet other recent studies confirm the original equalization hypothesis. This inconsistency represents a major challenge for research on stratification and mobility: the field cannot reach a conclusion or consensus over a core empirical question – whether family background does or does not affect the post-college mobility of graduates. In what follows we address this question in two ways: empirically by bringing new and arguably superior data to bear on the question, and theoretically, by considering measures, conceptualizations, and methodological issues that may generate the lack of consensus.

### 2.3 Parental background

How does parental background (social origin) influence offspring’s destination far beyond childhood? As discussed before, answering this question typically begins with a debate over how to measure hierarchical positions in the social structure (Breen & Jonsson, 2005). In the U.S. context, this debate has been recently expressed in a series of empirical studies that either show the relevance of breaking down ‘big classes’ into occupational micro classes (Weeden & Grusky, 2005; Weeden & Grusky, 2012) or argues for a resurrection of big classes because between-class income differences have grown in recent decades (Wodtke, 2017; Zhou & Wodtke, 2019). Nonetheless, it is important to note that, in the study of social mobility, researchers should be concerned with the extent to which social origin captures parental *resources* transmittable to the next generation through various forms of parental capital (economic, social, and cultural).

Several recent US studies have moved away from the occupational basis of social hierarchy and have focused instead on the resources available within childhood households. As argued by Atkinson (2010); Savage et al. (2014), Laurison and Friedman (2016); Friedman and Laurison (2019), occupational access is a reasonable single measure of class, but one that inadequately captures the effects – or mechanisms – of social origin on the next generation. In other words, offspring’s chances of success in education and thereafter are directly stratified by mechanisms rooted in parental income, parental wealth, or parental education.

Qualitative sociologists have documented several ways in which these different forms of capital affect the challenges and hurdles faced by children from lower-class backgrounds who are upwardly mobile (Friedman, 2016; Lareau, 2015; Rivera, 2015; Stuber, 2015). Even after successful completion of educational careers (e.g. college graduation), parental resources matter through ‘parental bridging’ (Armstrong & Hamilton, 2013): affluent parents are able to actively help new graduates in transitioning from college to their first job by paying off loans or by subsidizing their living expenses (e.g. rent) while they seek good jobs. This is not only a function of parental affluence (income or wealth) but also reflects an important cultural capital component (Rivera, 2015), including having college-educated parents who have experienced such transitions themselves.

Laurison and Friedman (2016) have recently quantified the influence of class-rooted capital far beyond college graduation. Using data from the United Kingdom, they document that children from working-class backgrounds who beat all odds and are able to enter elite jobs, do so with different resources and therefore do not achieve the same level of success. Using data from Britain, they attribute about 30 percent of the class pay gap to the *post-elite job* sorting mechanism, such as entering bigger firms or working in London. The authors call this the “class ceiling” effect.

### 2.4 Selection and equalization

Another approach to the impact of a college degree on destinations is to estimate the *relative equalizing* effect of college education vis-à-vis high school graduates – the difference in origin-destination association between the former and the latter. In order to test this, one has to account for selection (bias) of graduating college. Brand and Xie (2010) found that, after propensity score matching on a range of individual-level factors that determine college attendance, those who are least likely to graduate from college in fact benefit the most from it.^3^ This was termed *negative selection bias*. The importance of this study is that it demonstrated the mobility-enhancing function of the US educational system

Recently, Zhou (2019) revisited this by examining the extent to which intergenerational income mobility among college graduates differs from that of non-graduates using the NLSY’79. After accounting for selection processes – i.e. ‘treatments’ of varying levels of educational attendance – with a reweighting technique, Zhou’s study found that the origin-destination relationship is surprisingly similar for college graduates and non-college graduates. Further analyses showed a parental background gradient on children’s relative position (rank-rank approach) for both graduates of selective and nonselective colleges before reweighting. After reweighting, attending a selective college appeared have an equalizing effect, but it remained statistically non-significant compared to the nonselective college estimate.

Zhou (2019) argued that one should distinguish between selection effects, comparisons of mobility associations between educational levels, from equalization effects net of a particular educational pathway. The former assess the total impact of the educational *system* – designed with different levels and institutions – on intergenerational mobility, where the counterfactual of a graduate’s mobility is that same graduate’s mobility in case of not making it to the bachelor’s degree. The equalization effect, however, is understood as the ‘meritocratic power of a college degree.’ It asks whether a graduate of lower-class background reaches the same position as a graduate of a higher-class background. Its answer is straightforward, as college education largely is a meritocratic process, *conditional on selection processes* the effect of a disadvantaged parental background on offspring’s income shrinks to a non-significant gap.

In other words, studies on the *equalizing* effect of college education have to be concerned with selection into college, while studies testing the meritocratic thesis are concerned with *equalization* among one subgroup: college graduates. Despite being only one component of the entire institutional effect of higher education in intergenerational mobility – Zhou’s (2019) research question – the equalization thesis as formulated by Hout (1988) makes an assumption about the bachelor’s degree-holding population: implying a null relationship for social origin on destination status. Importantly, this is a testable hypothesis on nationally representative samples of bachelor’s degree holders; the analytical task of the current study.

## 3 Analytical approach and data

### 3.1 Research questions

In this paper, we revisit the question of persistence of family background influence on offspring’s labor market position among higher-educated Americans. Social mobility research has examined a variety of specific family background measures, including occupational standing, social class, income, wealth and education. Each of these measures is generally perceived as a *proxy* for the more complex ‘socio-economic origin’ concept because occupation is correlated with family earnings, wealth, IQ, cultural capital, attitudes and educational and career ambitions. However, we believe that parental education and parental income are more precise indicators of the mechanisms that explain intergenerational persistence of inequality. Following Torche’s (2011) comprehensive analysis of the meritocratic power of the college degree using a variety of measures origin and destination and subsequent assessments of equalization among college graduates in the 2010s, we consider erasing the effect of family background on socioeconomic destination as a testable hypothesis of social mobility for this educational subgroup.

Beyond this, our study is a step forward on at least one important empirical dimension. Several of the studies cited above rely on the NLSY or otherwise small-N datasets. By the time a working sample is selected consisting of parent-offspring pairs with the latter being bachelor’s degree holders, the sample size has dropped to about one thousand cases, or even a few hundred when split by selectivity level or terminal bachelor’s degrees. A small N can lead a null finding, a lack of association between parental background and offspring’s position, due to low statistical power. Hence, one motivation for our study is to overcome possible type II errors – false negatives – by obtaining a sample that contains many tens of thousands of college graduates with information on their parental background.

In our analyses of the relationship between family origin and adult offspring’s career position in the United States, we use very large samples of bachelor’s degree holders to answer the following three questions, each connected to a hypothesis about social mobility.

(1) What is the intergenerational association between parent’s highest education and offspring’s post-college earnings? This estimation addresses the equalization hypothesis. If educational attainment and occupational positions are (more) closely tied to measures of achievement rather than to ascription, then social origin or family background should no longer matter for offspring’s relative position *among* the higher-educated population. More specifically, a college degree should therefore erase social origin effects on earnings.
(2) Has the association between parental background and offspring’s earnings changed in recent years? Using sequential similarly harmonized datasets we compare the earnings advantage (and disadvantage) associated with parental education over time, covering birth cohorts from 1938 thru 1985. If the influence of social origin has substantially declined over time, this would be supportive of the liberal hypothesis. If the social origin effect remains the same over time, this would be supportive of the reproduction hypothesis.
(3) Do social origin advantages reappear or appear as a stronger influence among the youngest cohorts of advanced degrees holders (i.e., beyond the baccalaureate)? Here we take as our hypothesis Torche’s (2011, 2018) finding of a U-shaped origin-destination relationship where parental background re-emerges for post-baccalaureate holders.

We also perform a robustness check to see whether social origin is completely mediated by access to more selective colleges using a different nationally representative dataset. This issue is important given the evidence of both selection (by social origin) into different college levels (Espenshade & Radford, 2009; Ford & Thompson, 2016; Shavit, Arum, & Gamoran, 2007; Teichler, 2007), as well as the different pay-off levels of college selectivity or college quality (Brewer, Eide, & Ehrenberg, 1999; Thomas & Zhang, 2005; Witteveen & Attewell, 2017a; Zhang, 2005). We test whether the advantages associated with parental background (higher income in this case) hold across and within four-year college tiers. We address the possibility that college tier selection is completely driving findings regarding the origin-destination relationship among graduates.

Finally, we discuss issues of gender and race within intergenerational transmission. While many origin-destination studies focus on the father-son association of socioeconomic standing, our study examines includes both genders for parents and offspring. Recent mobility research – across educational groups – has also shown variation in the strength of the intergenerational mobility association for different racial groups (Chetty, Hendren, Jones, & Porter, 2018). These studies indicate substantially lower rates of upward mobility for blacks and higher rates of upward mobility for Hispanics, relative to whites. We will evaluate whether a similar trend is observable with regard to intergenerational effects on post-college destinations.

### 3.2 Estimation

Eq. (1) reflects the most elaborate model used in this study. Offspring’s annual logged earnings (W) is predicted by an ordinal (six category-) measure of highest parental education ($X_{i})$, that is used to estimate the slope coefficient of each level of parental education ($\beta_{1-6})$, vis-à-vis the reference category: parents who have a high school diploma as their highest education completed. These estimates are adjusted for a vector of pre-college ascription characteristics, F (gender, race/ethnicity, age, foreign-born). As parent’s education is associated with offspring’s own educational attainment^4^, we will present models that further control for this sorting mechanism. Vector $C_{i}$ reflects the influence of offspring having completed a bachelor’s degree only, a master’s degree, a doctorate, or a professional degree (ϕ).

(1) $$W_{i}=X_{i}\beta_{1-6}+F_{i}\gamma +C_{i}\phi +\varepsilon_{i}$$

We chose a negative binomial (NB) regression as the preferred functional form because of over-dispersion in the outcome variable (respondent’s earnings). Statisticians have convincingly argued that merely predicting the natural log of earnings using an OLS regression – a popular alternative – does not sufficiently account for outcome distributions that are heavily skewed to the right (Hardin & Hilbe, 2012; Hilbe, 2011, 2014). When comparing the log-likelihood of a Poisson regression and a NB regression, we find substantially larger (less negative) log-likelihoods for the latter, confirming a better model fit. Our substantive findings are not limited by this modeling decision, as discussed in a robustness section.

### 3.3 Data

We analyze nationally representative data from the U.S. National Survey of College Graduates (NSCG) (NSF 2015), undertaken by the National Science Foundation. It contains of a series of cross-sectional surveys, with bachelor’s degree holders (or higher), drawn from the American Community Survey (ACS). To highlight consistency or change over time, we use the NSCG surveys of 1993 (N = 148,905), 2003 (N = 100,402), and 2015 (N = 91,000), each representing the population of bachelor’s degree holders in the United States in the respected observation year.^5^

We selected a study sample of employed individuals between ages 30 and 55 to minimize the influence of late labor market entry and early retirement. We also exclude individuals who are still enrolled in education in the reference year. In case of missing information on parental education we listwise-deleted these records from the analyses (about one percent of the samples). Appendices B and C present our main findings with Heckman-corrected estimates for selection bias. Our final study sample contains 190,765 records linking family background to offspring’s earnings for college-educated Americans, across the three observation years: 1993 (88,721), 2003 (58,179), and 2015 (43,856).

The key dependent variable is the annual salary of respondents. This variable was top-coded by NSF at $150,000 in 2015 survey. We applied the same top-coding to the 1993 and 2003 data. For parental education we use the highest educational level completed by either parent, creating an ordinal variable containing categories for less than high school (1), high school diploma (2), some college or associate’s degree (3), bachelor’s degree (4), master’s degree (5), and doctorate (6). However, the 1993-version recorded parent’s higher education attainment by bachelor’s degree completion vs. ‘attended graduate school,’ without distinguishing between master’s and doctorates.

The control variables are gender, age in the reference year, race/ethnicity (white, black, Hispanic, Asian, Native American, and other), and a dummy for whether the respondent was foreign-born. We use the respondent’s own higher education attainment (BA only, MA degree, doctorate, or professional degree) as an additional control in some models, and in some models we split the analyses by offspring’s highest degree obtained (BA only vs. advanced degree).

For our robustness checks regarding college tiers, we make use of the cross-sectional statistics by college tier and parent income percentile derived from the aggregated public data by Chetty et al.’s (2017) study of ‘The Role of Colleges in Intergenerational Mobility.’^6^ The original data for parents and their children came from the Internal Revenue Service (IRS). The data derived for the Chetty’s mobility project are a pooled sample of individuals from 1980 thru 1982 birth cohorts, measuring the current incomes of about 10 million former college attendees who are in their mid-thirties in 2015, as well as their parents using previous waves of IRS records. We select nine four-year college tiers as used in this dataset: Ivy League plus, other elite schools (public/private), highly selective public, highly selective private, selective public, selective private, nonselective four-year public, non-selective four-year private not-for-profit, and four-year for profit. Using OLS regressions and weighted-least-squares (WLS) regressions, we estimate the association between parental income and offspring’s income using group-mean structured data by percentile within college tier (9). This selection is representative of 3.6 million former college attendees (both graduates and non-graduates).

## 4 Findings

### 4.1 Earnings differences associated with parental education

We present the main findings in Table 1. For each survey year (1993, 2003, and 2015), we first estimate the relationship between parental education and offspring’s earnings for the working population between ages 30 and 55. The reference group for parental education is having completed a high school degree. The table reports incidence-rate ratios (IRRs) from negative binomial regressions converted into percentages to aid interpretation: a coefficient of -.044 should be read as 4.4 percent lower earnings. Each survey year’s leftmost model – titled ‘baseline’ – reports the relationship between parental education and offspring’s annual earnings after controlling for age, foreign born, race/ethnicity and gender. Given space considerations, the coefficients of most control variables are omitted from Table 1 but are fully reported in Appendix A.^7^

**Table 1:** Parental Education Estimates on Earnings (IRR-% conversions from NB regressions).

	1993		2003		2015	
	baseline	+ highest degree	baseline	+ highest degree	baseline	+ highest degree
**parental education** (ref = HS)						
less than high school	−.044***	−.035***	−.024	−.025	−.042	−.040
	(.006)	(.006)	(.016)	(.016)	(.040)	(.039)
some college / associate’s	.010	.003***	.029*	.012	.014	−.003
	(.007)	(.007)	(.013)	(.013)	(.029)	(.028)
bachelor’s	.079***	.059***	.131***	.098***	.099**	.066*
	(.008)	(.008)	(.015)	(.014)	(.031)	(.029)
master’s			.135***	.062***	.159***	.100**
			(.018)	(.016)	(.037)	(.035)
doctorate			.247***	.089***	.308***	.119**
			(.021)	(.019)	(.046)	(.036)
graduate school attainment	.112***	.034***				
	(.008)	(.007)				

**female**	−.270***	−.249***	−.368***	−.359***	−.325***	−.327***
	(.004)	(.004)	(.006)	(.006)	(.012)	(.011)

**summary statistics**						
N	88,721	88,721	58,179	58,179	43,865	43,865
alpha	.244	.222	.486	.456	.472	.444

*Note.* The baseline model also adjusts for age group, foreign born, and race / ethnicity. The highest degree model adds individuals’ post-graduate completion (MA, doctorate, professional degree) to the model. The coefficients of the control variables are reported in Appendix A. Robust standard errors between parentheses. Sampling weights are applied. IRR = incidence-rate ratios, which are converted to a -1 to 1 range to reflect percent-effect sizes. P-values: *** <.001, **<.01, * = <.05 (two-tailed tests).

Focusing on parental education *advantages*, children of parents with a bachelor’s degree earn about 7.9 percent more (in 1993), 13.1 percent more (in 2003), and 9.9 percent more (in 2015), compared to counterparts whose parents had high school diplomas. Although the estimates of parental education fluctuate somewhat in size across these three cross-sectional surveys that span 22 years, they are all substantial and statistically significant.

The second column in Table 1 adds offspring’s highest degree to the previous model. Clearly some of the earnings advantage of those with highly educated parents is attributable to more of their offspring completing higher degrees, compared to children of less educated parents. This is evident from the fact that the coefficients for parental education in this second column are smaller than the equivalent coefficients in the baseline model. However, even after controlling for highest degree attained by offspring in the model reported in the second column of Table 1, the parental education coefficients are still substantial and statistically significant. Controlling for offspring’s educational attainment reduced the earnings advantage of parents with a bachelor’s degree by about one-third.

Even larger earnings advantages are found when parents had higher degrees. Respondents with parents with a master’s (or equivalent) earned 13.5 percent more (2003) to 15.9 percent more (2015) – and those whose parents had a doctorate earned 24.7 percent more (2003) to 30.8 percent (2015). In the 1993 survey, with a combined higher degree category, offspring of parents with higher degrees earn about 11.2 percent more than graduates with high school educated parents. To summarize: some of the parental influence of having a parent with a higher degree (post-baccalaureate) operates through their offspring also attaining higher degrees, which reduces the parental coefficient by about two-thirds. Yet the direct effect of parental education remains substantial and statistically significant.

### 4.2 Offspring’s level of higher education

Table 2 provides a parallel analysis to Table 1, but here the estimates are fitted on subsamples of offspring having different levels of higher education attainment: bachelor’s degree only and master’s degrees or higher (including PhD’s and professional degrees). We do this in the light of Torche’s (2011) argument that parental background affects outcomes for offspring with higher degrees but not for those who only complete a baccalaureate. In Table 2, among those who did not go further than a bachelor’s degree, the intergenerational advantage or disadvantage associated with parental education is still evident. Having parents with bachelor’s degrees, master’s degrees, or doctorates (or graduate school attainment) is associated with significantly higher offspring’s earnings, compared to parents who were high school graduates. The estimates are smaller in 1993: 5.5 percent higher earnings for parental BA’s and 3.5 percent for parental graduate school attainment. By the 2003 and 2015 surveys, the parental BA advantage was around 10 percent, while having parents with post-graduate degrees yielded even larger advantages (especially in the 2015 survey). This contradicts the equalization hypothesis cited earlier that parental education is not significantly related to earnings among offspring with only a bachelor’s degree.

**Table 2:** Parental Education Estimates on Earnings by Highest Educational Level (IRR-% conversions from NB regressions).

	bachelor's only			advanced degrees only		
	1993	2003	2015	1993	2003	2015
**parental education** (ref = HS)						
less than high school	−.029***	−.020	.010	−.058***	−.017	−.128*
	(.008)	(.022)	(.057)	(.010)	(.024)	(.048)
some college / associate’s	.007	.002	.021	.010	.065**	−.041
	(.009)	(.016)	(.035)	(.011)	(.020)	(.046)
bachelor's	.055***	.095***	.100**	.092***	.156***	.035
	(.010)	(.018)	(.037)	(.012)	(.025)	(.047)
master's		.061**	.160***		.127***	.043
		(.023)	(.049)		(.025)	(.051)
doctorate		.094***	.128*		.245***	.246***
		(.028)	(.054)		(.028)	(.063)
graduate school attainment	.035**			.105***		
	(.011)			(.012)		

**female**	−.263***	-.374***	−.332***	−.279***	−.363***	-.337***
	(.005)	(.007)	(.015)	(.005)	(.009)	(.016)

**summary statistics**						
N	51,546	31,706	19,307	37,175	26,473	24,558
alpha	.228	.487	.464	.241	.441	.443

*Note.* All models also adjust for age group, foreign born, and race / ethnicity. Post-graduate degrees include masters, doctorates, and professional degrees. Robust standard errors between parentheses. Sampling weights are applied. IRR = incidence-rate ratios, which are converted to a -1 to 1 range to reflect percent-effect sizes. P-values: *** <.001, **<.01, * = <.05 (two-tailed tests).

In the separate estimates of parental education on the earnings of respondents who had obtained a masters or higher in Table 2, individuals with college-educated parents earned at least 9.2 percent more than their counterparts with high school-educated parents (1993). That earnings advantage was even larger for children of PhD graduates (roughly 25 percent in 2003 and 2015).

### 4.3 Life course effects

Three cross-sectional surveys each indicated a substantial relationship between parental education and offspring’s mid-career earnings for college-educated individuals aged between 30 and 55. However, it is conceivable that this intergenerational association could partly reflect one’s career phase. To examine that possibility, we ran separate models for three career phases in each of the three surveys: ages 30–39, ages 40–49, and ages 50–55.

Reading the upper panel in Table 3 – the youngest age group (30–39) – from left to right, one observes a quite consistent earnings advantage for college graduates whose parents are more highly educated (BA, MA, doctorate, graduate school attainment). Among employed individuals in their thirties with bachelor’s degree parents, all earned more than their counterparts with high school educated parents, *regardless* of their varying birth cohorts (1954–1963, 1964–1973, 1976–1985). These coefficients translate to earnings advantages of 7.6 percent, 13.7 percent, and 12.6 percent, respectively.

**Table 3:** Parental Education Estimates on Earnings by Age Group (IRR-% conversions from NB regressions).

	1993	2003	2015
**parental education** (ref = HS)			
	Birth Cohorts		
**ages 30–39**	'54–'63	'64 - '73	'76 - '85
<high school	−.044***	.003	−.065
	(.011)	(.033)	(.073)
some college / associate's	−.003	.015	.031
	(.011)	(.023)	(.044)
bachelor's	.076***	.137***	.126**
	(.012)	(.025)	(.044)
master's		.144***	.163***
		(.025)	(.050)
doctorate		.263***	.372***
		(.036)	(.072)
graduate school attainment	.104***		
	(.012)		
	Birth Cohorts		
**ages 40–49**	'44–'53	'54–'63	'66–'75
<high school	−.048***	−.030	−.096
	(.009)	(.026)	(.055)
some college / associate's	.021*	.045*	.009
	(.011)	(.020)	(.049)
bachelor's	.089***	.117***	.091
	(.013)	(.024)	(.053)
master's		.126***	.157**
		(.031)	(.064)
doctorate		.221***	.324***
		(.032)	(.074)
graduate school attainment	.127***		
	(.014)		
	Birth Cohorts		
**ages 50–55**	'38 - '43	'48 - '53	'60 - '65
<high school	−.035*	−.022	.044
	(.015)	(.027)	(.079)
some college / associate's	.009	.033	.003
	(.019)	(.026)	(.057)
bachelor's	.081***	.161***	.082
	(.023)	(.033)	(.060)
master's		.135***	.169*
		(.040)	(.082)
doctorate		.270***	.175*
		(.046)	(.087)
graduate school attainment	.103***		
	(.023)		
**NSCG sample N**	**88,721**	**58,179**	**43,865**
ages 30–39	37,658	20,251	21,861
ages 40–49	37,771	24,229	13,410
ages 50–55	13,292	13,699	8,594

*Note.* All models also adjust for gender, foreign born, and race / ethnicity. Robust standard errors between parentheses. Sampling weights are applied. IRR = incidence-rate ratios, which are converted to a -1 to 1 range to reflect percent-effect sizes. P-values: *** <.001, **<.01, * = <.05 (two-tailed tests).

Similarly, the second panel of Table 3 repeats the analysis for individuals in their forties in each of the survey years; reflecting the estimates for slightly older birth cohorts: 1944–1953, 1954–1963, and 1966–1975. Again, the earnings boost is substantial for offspring of bachelor’s degree-holding parents, ranging from 8.9 percent to 11.7 percent higher earnings. The earnings advantages for individuals with parents with advanced degrees also remain large in comparison to the reference group (parents with a high school diploma) for the 40–49 age group.

Among the oldest employed individuals observed in this study – aged 50–55 – there is also an intergenerational association between parental education and offspring’s earnings. For instance, in Table 3 among the oldest birth cohort in our study (1938–1943) having parents with a bachelor’s degree or graduate school attainment is associated with 8.1 percent higher earnings and 10.3 percent higher earnings, respectively, when the offspring were in their fifties. The single exception is the 2015 observation of employed individuals between 50 and 55. Among this subgroup, parental education of a bachelor’s degree (only) is no longer significantly associated with an earnings advantage vis-à-vis parental education of just a high school degree.

We conclude that the estimates reflecting persistence of inequality through parental education, affecting offspring’s earnings, are *not* dependent on an individual’s career phase. On the contrary, the coefficients for parental education advantage are remarkably stable across the life course. Equally important, this association between family background and offspring’s destination has been there for a long time. Many of the birth cohorts in our data overlap with Hout’s studies (1984, 1988), who selected employed individuals aged 25 thru 64 between 1962 and 1973 and between 1972 and 1985.

### 4.4 Gender and race

All of our previous models show that college-educated women earn substantially less than men; the gender earnings gap ranges between a 25 percent and a 37 percent disadvantage for women, net of controls. Since gender is such an important determinant of earnings among the college-educated, we estimated separate intergenerational models for men and women, presented in Table 4.

**Table 4:** Parental Education Estimates on Earnings by Gender (IRR-% conversions from NB regressions).

	1993		2003		2015	
	men	women	men	women	men	women
**parental education** (ref = HS)						
<high school	−.050***	−.036***	−.044*	−.004	−.101	.014
	(.008)	(.010)	(.021)	(.025)	(.055)	(.058)
some college / associate's	.006	.015	.046*	.010	-.018	.041
	(.009)	(.011)	(.019)	(.018)	(.041)	(.041)
bachelor's	.099***	.056***	.162***	.093***	.093*	.103*
	(.010)	(.013)	(.020)	(.023)	(.043)	(.042)
master's			.154***	.113***	.099*	.213***
			(.023)	(.027)	(.045)	(.057)
doctorate			.322***	.156***	.305***	.313***
			(.032)	(.027)	(.062)	(.068)
graduate school attainment	.115***	.113***				
	(.011)	(.014)				
**summary statistics**						
N	56,688	32,033	34,068	24,111	23,667	20,198
alpha	.256	.220	.425	.551	.413	.520

*Note.* All models also adjust for age group, foreign born, and race / ethnicity. Robust standard errors between parentheses. Sampling weights are applied. IRR = incidence-rate ratios, which are converted to a -1 to 1 range to reflect percent-effect sizes. P-values: *** <.001, **<.01, * = <.05 (two-tailed tests).

The robustness checks performed with the NSCG data reports both significant and substantial earnings advantages for children of higher educated parents across both genders and for all three survey years. While the 1993- and 2003-observations displayed a slightly larger parental background association for men than for women, the 2015-observations indicates a rather similar pattern between both genders. Having parents with a bachelor’s degree yields about 10 percent higher earnings compared to families with a high school diploma only, for both men and women (most-right columns). Likewise, the advantage of PhD-level educated parents is the same (31 percent) for both genders. The advantage of master’s degree-holding parents is larger for women than for men.

In addition, we estimated separate models for whites, blacks, Hispanics, and Asians to assess whether our test of the meritocratic thesis regarding post-college destinations differs for different racial groups. Table 5 presents an exact replication of our baseline estimates from Table 1 for all four racial groups, which include controls for age group and immigrant background. As shown here, all racial groups, across all three observation years, display substantive significant associations between parental education and offspring’s earnings. Expanding the models with an additional control for respondent’s highest degree attained influences the associations in a similar fashion as in our main model (therefore not shown). While the sizes of the associations fluctuate somewhat between observation years, we conclude that our substantive finding – disproving equalization among college graduates – holds for all four racial groups.

**Table 5:** Parental Education Estimates on Earnings by Race (IRR-% conversions from NB regressions).

	1993			
	white	black	Hispanic	Asian
**parental education** (ref = HS)				
<high school	−.045***	−.042**	−.079***	−.004***
	(.007)	(.014)	(.019)	(.019)
some college / associate's	.006	.049*	.010	.043*
	(.008)	(.020)	(026)	(.021)
bachelor's	.081***	.084**	.075*	.077***
	(.009)	(.031)	(.031)	(.021)
graduate school attainment	.105***	.241***	.109**	.187***
	(.009)	(.034)	(.035)	(.026)

	2003			
	white	black	Hispanic	Asian
**parental education** (ref = HS)				
<high school	.018	−.115***	−.133***	.052
	(.025)	(.031)	(.034)	(.042)
some college / associate's	.028	.038	−.021	.110**
	(.015)	(.033)	(.040)	(.043)
bachelor's	.139***	.094*	.072	.147***
	(.018)	(.041)	(.049)	(.038)
master's	.124***	.110**	.212***	.277***
	(.020)	(.044)	(.062)	(.053)
doctorate	.239***	.329***	.271***	.246***
	(.025)	(.080)	(.070)	(.050)

	2015			
	white	black	Hispanic	Asian
**parental education** (ref = HS)				
<high school	−.147*	−.037	.071	.015
	(.068)	(.722)	(.084)	(.065)
some college / associate's	.010	.080	.084	−.079
	(.035)	(.448)	(.088)	(.078)
bachelor's	.116**	−.039	.050	.090
	(.038)	(.694)	(.085)	(.065)
master's	.136**	.202*	.279**	.252**
	(.043)	(.144)	(.113)	(.084)
doctorate	.287***	.338*	.636**	.326***
	(.054)	(.047)	(.196)	(.096)

*Note.* Replication of Table 1 (baseline model). Robust standard errors between parentheses. Sampling weights are applied. P-values: *** <.001, **<.01, * = <.05 (two-tailed tests).

However, the 2015 estimates for blacks, Hispanics, and Asians deviate slightly from the two previous observation years (and also from whites). For these three racial/ethnic minorities, the intergenerational impact of parental education on earnings is concentrated in the upper levels of parental higher education – vis-à-vis high school – rather than the parental bachelor’s degree. This could be considered a trend towards a specific kind of intergenerational impact on post-college earnings *among* blacks, Hispanics, and Asians, whereby only the minority children from the most privileged backgrounds experience an earnings advantage from their parental background.

### 4.5 Selection bias and functional form specification

The analyses presented above are conditional on a respondent being employed and not being enrolled in education during the survey year. We examined potential selection bias initially by running a logistic regression predicting selection into the analysis sample using the full set of predictors. The odds ratios for the parental education predictor and gender are presented in Appendix B. They show that offspring from higher-educated families are less likely to be in the study sample, especially for the 1993 and 2003 survey years. Women are also consistently less likely to be employed (and not enrolled) than men.

To overcome potentially biased estimates resulting from selection, we applied a two-stage Heckman correction to our baseline negative binomial regressions (Cameron & Trivedi, 2010; Heckman, 1977). We report the IRR (percent-converted) estimates for parental education and for gender on earnings from these new models in Appendix C. Some of coefficient sizes shifted a little, both positively and negatively, but the earnings advantages associated with having higher-educated parents remain statistically significant in almost all models.

Finally, we chose the negative binomial regression instead of the commonly used OLS regressions on logged earnings because a logarithmic transformation does not sufficiently account for over-dispersion. Statisticians such as Hilbe (2014) explicitly warn against using linear regression with a logged count variable as sufficiently addressing overdispersion, although this was the normal methodology in many of the papers reviewed earlier. As a check, however, we tested whether using logged earnings as the dependent variable would lead to a different conclusion regarding parental background advantage. The magnitude of coefficients changed slightly but none of the estimates’ p-values changed.

### 4.6 Within and across college tiers

**Fig. 1:**
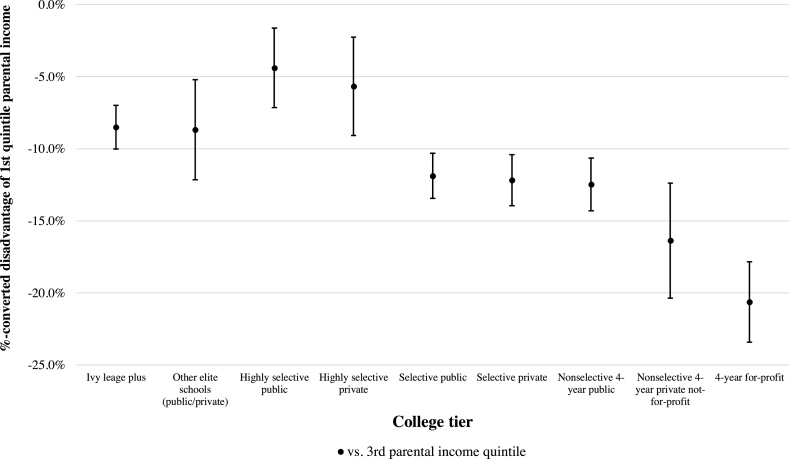
Earnings Disadvantages for Children from the Lowest Parental Income Quintile vis-à-vis the Middle Quintile by College Tier. *Note:* The model regresses aggregate parental income percentile (by college tier) on aggregated logged offspring’s income in 2015 in a weighted-least-squares regression. Individuals are born in 1980, 1981, and 1982 (average 34 years of age). The error bars reflect a 95 %-confidence interval.

*Source:* Authors’ calculations of ‘Table 6. Cross-Sectional Statistics by College Tier and Parent Income Percentile’ data from Chetty et al. (2017): Mobility Report Cards: The Role of Colleges in Intergenerational Mobility.

Next, we turn to our analyses concerning college tier selection. Fig. 1 presents the estimated earnings *disadvantage* of children from the lowest parental income quintile (about $16,000) vis-à-vis their counterparts from middle-quintile income families (about $61,000) separately for the nine college tiers as identified by Chetty et al. (2017). These estimates are based on the logged 2015 incomes of individuals in their mid-30 s (transformed to percent gaps).

We observe substantially larger within-college-tier earnings disadvantages for students from the poorest families compared to the ‘average college student.’ Being from a bottom-quintile income family is associated with a 20.4 percent earnings deficit within the four-year for-profit tier and a 16.4 % deficit within the nonselective four-year private college. An equivalent low-income family origin seems to reduce post-college earnings slightly less within selective public colleges (−11.9 percent), selective private colleges (−12.2 percent), and non-selective four-year public colleges (−12.5 percent). With exception of the overlapping confidence intervals with ‘other elite schools,’ the most selective college tiers display significantly lower within-tier earnings disadvantage for students from the lowest parental income quintile. The deficit compared to the middle-parental quintile group is between 4.4 percent and 8.7 percent.

**Fig. 2:**
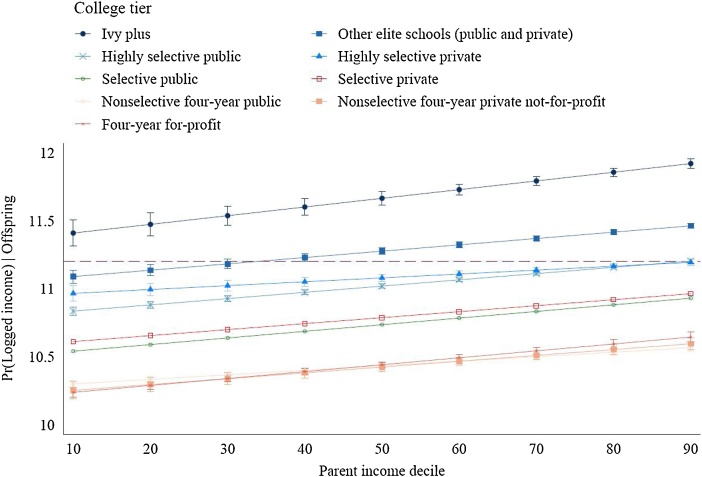
Marginal Effects on Offspring’s Income by College Tier*Parental Income Interaction. *Note.* The model regresses aggregate parental income percentile (by college tier) on aggregated logged offspring’s income in 2015 in a weighted-least-squares regression. The graph plots the marginal intergenerational income association from a college tier*parental income interaction. Individuals are born in 1980, 1981, and 1982 (average 34 years of age).

*Source.* Authors’ calculations of ‘Table 6. Cross-Sectional Statistics by College Tier and Parent Income Percentile’ data from Chetty et al. (2017): Mobility Report Cards: The Role of Colleges in Intergenerational Mobility.

Following Zhou’s (2019) approach with regarding to moderation of the college selectivity level, we then ran an interaction model for the combined college tier and parental income association of offspring’s earnings. Adjusted for the main associations of college tier and parental income, Fig. 2 presents the margins on the logged earnings of offspring for parent income deciles by college tiers. We first notice the (expected) order of earnings levels by college tiers, from Ivy League at the very top to non-selective four-year private and four-year for profit at the bottom of the incomes-estimate hierarchy. As this interaction measures the influence of parental income across college tiers, the fact that each college tier line indicates a significant (95 %-CI’s) upward slope suggests that low parental income disadvantages throughout the college-educated population.

In sum, there is little doubt that college tiers *reduce* the origin-destination association (moderation), at least among mid-career individuals in 2015. Generally speaking, more selective college tiers not only yield a higher income potential, they also contain less of a family background disadvantage. However, regardless of college tier, a lower-income family background still remains significantly associated with lower average post-college earnings.

## 5 Conclusion and discussion

Analyzing three different cross-sectional surveys of college graduates – from 1993, 2003 and 2015 – we observed a consistent pattern whereby greater parental education was associated with substantially higher earnings for baccalaureate offspring, and lower parental education was associated with lower offspring’s earnings. This parental transmission of advantage occurred for graduates with a bachelor’s degree, as well as for individuals with advanced degrees. Together, these findings contradict the equalization hypothesis: A college degree may partly equalize, but it is not nearly enough to erase the influence of social origin. In short, by achieving a college degree an individual does *not* escape their social origins, according to these analyses, which are in accord with other several recently published papers that use other datasets and measures.

Modernization theory predicted that, as the meritocratic system expanded through more access to higher education and more occupational selection was based on educational credentials rather than ascription, social origin would matter less for younger cohorts and ultimately be delinked. On the contrary, we found that the pattern of social origin associations did not change much across the three surveys that spanned birth cohorts from 1938 to 1985, not in significance levels, but also not in terms of effect sizes. We believe that this persistence of parental background effects is in line with the broader reproduction (or ‘constant flux’) hypothesis about shifts in social mobility over time. Despite increasing access to higher education over time, the intergenerational association is surprisingly stable across older and younger cohorts and is evident for men and for women, and for ethnic/racial minorities.

To be sure, more recent economics research (e.g. Chetty et al., 2017) and sociological research by Karlson (2019); Torche (2011), and others, have expanded the scope of the equalization thesis to newly available measures of graduates’ destination, including their SES, family income, and earnings. These studies have all been published in the past ten years and are partially rooted in better (income) data becoming available to researchers through panel studies and administrative records. Using income or (family) earnings as destination allows for a much more detailed indication of socioeconomic standing and thereby a significant step forward in terms of accuracy. Compared to annual earnings, as used in this study, the more traditional occupational prestige measures group together jobs in occupational classes with varying earnings levels. That procedure ignores a major source of variation in socioeconomic standing. Nonetheless, a substantive number of studies have tested the *same* equalization thesis using this variety of measures. This study followed in that tradition.

The primary purpose of this study was to assess a testable hypothesis regarding social origin using recent, high quality, and large N datasets. Strengthening the statistical power and using multiple waves of data from the National Surveys of College Graduates yielded results that are not consistent with Hout’s (1984, p.1404) earlier argument that “origin status does not affect destination status among college graduates.” Our findings challenge Torche’s (2011, p.798) claim that “the chances of achieving economic success are independent of social background among those who attain a BA. The finding is largely consistent across all indicators of socioeconomic standing…” The advantages and disadvantages associated with parental background we report for the NSCG echo results from several recent papers using different nationally representative datasets, as reviewed earlier (e.g. Emmons et al., 2018; Ford, 2018; Manzoni & Streib, 2018). Furthermore, our findings are also consistent with the conclusions of several other origin-destination studies, situated in similar higher education systems in comparable countries, such as the United Kingdom (Bukodi & Goldthorpe, 2019; Friedman & Laurison, 2019).

Hout’s original equalization thesis from the 1980s has since been expanded in at least two directions. First, Torche (2011) found a so-called U-shaped mediation of the origin-destination association whereby social origin effects ‘reappear’ among advanced degree holders. Our findings also indicated significant earnings advantages associated with offspring attainment of higher degrees. This is however not a phenomenon that ‘reemerges’ at the post-graduate education level because social origin effects remained significant (and large) among those with a terminal bachelor’s degree. As noted by Karlson (2019), a reemergence would also be at odds with the selectivity hypothesis, which states that among the most selected groups a stronger meritocratic sorting mechanism should decouple origin from destination. Still, as Karlson’s (2019) reported no effect of social origin on destination SES among both bachelor’s degree holders *and* advanced degree holders using the NLSY’97, our findings contrast with his study with regard to both levels of higher education.

Second, although less theorized in the literature, the selection into different college selectivity levels, which entails social origin gradients, may lead to varying levels of status reproduction. As argued by Chetty et al. (2017), attending a highly selective college reduces the intergenerational association to about one quarter of the total association. Although selection into college tiers of greater selectivity has a parental background gradient, one might assume that lower-SES graduates from the most selective institutions achieve upward mobility, thereby offsetting an intergenerational effect at that specific level. Thompson (2019) recently showed that this is not the case with regard to the origin incomes and destination wages *within* selective US institutions. Our NSCG analysis does not (and *cannot*) address the mechanism of within-college-tier mechanisms of intergenerational inequality, let alone the mechanisms within individual colleges. We find, however, that the overall intergenerational association of advantage/disadvantage is only partially mediated by college tier. There remains a direct effect of family background on offspring’s post-college earnings.

How can one reconcile the findings from our analyses with the earlier claim that by attaining a baccalaureate, a person escapes his or her family origins? The simplest explanation would be that the world has changed over time, and that stratifying processes have strengthened since the equalization thesis was first proposed. However, we do not consider that likely, because the three surveys analyzed here covered birth cohorts from 1938 to 1985 and the parental education association appeared throughout.

A more plausible explanation, in our opinion, is to be found in shifts in how social status is measured and conceptualized. The earlier status attainment tradition frequently used large occupational categories to represent origins (family background) and destinations (offspring’s attainment) when estimating log linear models of mobility and intergenerational transmission. Alternatively, an index was used, representing the income and educational of occupations. In contrast, the more recent literature cited above typically uses offspring’s income as a measure of destination. These two approaches yield different findings, in part because occupational categories are not the best predictors of lifetime income, as Kim, Tamborini, and Sakamoto (2018) have recently documented.

However, this argument does not account for the discrepancy with Torche’s (2011) study which did analyze personal income as one outcome. However, her null findings (for BA holders) came from longitudinal analyses of the PSID, where her reported sample sizes for parent-offspring pairs were very small: for example, only 177 men and 134 women were in the BA-only group. When sample sizes grow that small it becomes difficult to know whether a lack of statistical significance (a null finding) for a regression coefficient reflects a lack of statistical power, or is a true finding. The several studies that did find intergenerational transmission, both this NSCG study and some of those discussed earlier, had substantially larger sample sizes, which may explain why the coefficients that Torche reported were not statistically different from zero, but were significant in these other studies.

Finally, following recent studies by Chetty et al. (2017, 2018), who have reported racial variation, we tested whether the intergenerational impact of parental education on offspring’s earnings holds for whites, blacks, Hispanics, and Asians. This is the case. While Chetty et al. (2018) report lower *absolute* intergenerational mobility rates for black Americans across society, we cannot find evidence for a substantively different type of intergenerational persistence of advantage for blacks. However, our most recent observation hints at a concentration of intergenerational advantage for minority students from the highest parental educational backgrounds. This should not be confused with a disappearance of intergenerational inequality (or equalization) for racial/ethnic minorities. Future research should first concentrate on whether this trend persists and subsequently explore the possible over-time changes in parental status of racial/ethnic groups in order to interpret these variations.

## Declaration of Competing Interest

None.

## Figures and Tables

**Table:** 

	1993	2003	2015
**age group** (ref = 30–34)			
35 to 39	.142***	.124***	.153***
	(.008)	(.017)	(.029)
40 to 44	.196***	.233***	.234***
	(.009)	(.019)	(.034)
45 to 49	.260***	.250***	.296***
	(.009)	(.019)	(.034)
50 to 54	.309***	.214***	.343***
	(.011)	(.018)	(.037)
55+	.296***	.153***	.422***
	(.022)	(.030)	(.081)

**foreign born**	.030***	.031*	.106**
	(.007)	(.013)	(.033)

**race / ethnicity** (ref = white)			
black	−.103***	−.098***	−.103**
	(.006)	(.012)	(.034)
Hispanic	−.052***	−.120***	−.128***
	(.008)	(.013)	(.027)
Asian	−.022**	.027	.060
	(.008)	(.016)	(.033)
American native	−.145***	−.077*	−.120**
	(.018)	(.032)	(.041)

**Table:** 

	1993	2003	2015
**parental education** (ref = HS)			
<high school	.965	.937	.780
	(.031)	(.055)	(.129)
some college / AA	.883***	.954	1.067
	(.028)	(.043)	(.110)
bachelor's	.737***	.833***	.822*
	(.024)	(.036)	(.081)
master's		.793***	1.006
		(.040)	(.105)
doctorate		.765***	1.007
		(.042)	(.117)
grad. school attainment	.729***		
	(.023)		

**female**	.164***	.374**	.455***
	(.004)	(.011)	(.030)

**summary statistics**			
N	107,216	69,815	50,845
f (in study sample)	88,721 (82.7 %)	58,179 (83.3 %)	43,865 (86.3 %)
Wald Chi	6685.8	1231.0	198.5
prob.	.000	.000	.000

**Table:** 

	1993	2003	2015
**parental education** (ref = HS)			
<high school	−.038***	−.107***	−.096*
	(.006)	(.019)	(.040)
some college / associate's	.030***	−.020	.052
	(.008)	(.015)	(.032)
bachelor's	.128***	.068*	.128***
	(.010)	(.021)	(.032)
master's		−.038	.145***
		(.028)	(.037)
doctorate		.138***	.217***
		(.032)	(.046)
graduate school attainment	.174***		
	(.012)		

**female**	.006	−.681***	−.653***
	(.038)	(.033)	(.049)

**summary statistics**			
N	88,721	58,179	43,865
alpha	.244	.486	.470

## Appendix A Control Variable Estimates of the Baseline Earnings Models, Table 1 (IRR-% conversions from NB regressions)

*Note.* Robust standard errors between parentheses. Sampling weights are applied. IRR = incidence-rate ratios, which are converted to a -1 to 1 to reflect percent-effect sizes. P-values: *** <.001, **<.01, * = <.05 (two-tailed tests).

*Source.* National Survey of College Graduates 1993, 2003, and 2015 (selection of employed individuals between 30 and 55).

## Appendix B Odds ratios of Employment (and not enrolled) in the Reference Year of Earnings

*Note.* All models also adjust for age group, foreign born, and race / ethnicity. Robust standard errors between parentheses. Sampling weights are applied. P-values: *** <.001, **<.01, * = <.05 (two-tailed tests).

*Source.* National Survey of College Graduates 1993, 2003, and 2015 (all individuals between 30 and 55).

## Appendix C Heckman-corrected Parental Education Estimates on Earnings (IRR-% conversions from NB regressions)

*Note:* All models also adjust for age group, foreign born, and race / ethnicity. Robust standard errors between parentheses. Sampling weights are applied. P-values: *** <.001, **<.01, * = <.05 (two-tailed tests).

*Source:* National Survey of College Graduates 1993, 2003, and 2015 (selection of employed individuals between 30 and 55).

## Footnotes

1 Blau and Duncan (1967) confirmed Lipset and Bendix’s (1959) earlier conclusion that the US was not exceptional in its rate of mobility from manual to non-manual occupations. However, they argued that the US had exceptional amounts of mobility from the bottom to professional occupations. They also documented that parental background (their income and education) explained less than half of offspring’s outcomes, that offspring’s educational attainment accounted for the lion’s share of variance in their outcomes, and that the path from origin to destination was almost all indirect via offspring’s education.

2 Torche (2011) uses class, occupational status, earnings, and household income as origin measures.

3 See Breen, Choi, and Holm (2015) for a replication of this paper, as well as a discussion of selection bias and heterogeneous effects.

4 The Spearman correlations (r) between parent’s education and offspring’s education are .087 (1993), .1347 (2003), and .1615 (2015).

5 More information about the NSCG surveys can be found on: https://www.nsf.gov/statistics/srvygrads/.

6 More information about Chetty et al.’s (2017) mobility data can be found on: http://www.equality-of-opportunity.org/data/.

7 f we were to control further for one’s college major, the parental advantages are attenuated just slightly. For instance, for the 2015 model, a parental BA yields an advantage of .083 (p<.01) vis-à-vis the reference group if college major is added to the model, compared to a .099 (p<.01) association in the main model.
